# Optimizing radiation dose by varying age at pediatric temporal bone CT

**DOI:** 10.1120/jacmp.v16i1.5082

**Published:** 2015-01-08

**Authors:** Daichi Noto, Yoshinori Funama, Mika Kitajima, Daisuke Utsunomiya, Seitaro Oda, Yasuyuki Yamashita

**Affiliations:** ^1^ Department of Graduate School of Health Sciences Kumamoto University Kumamoto Japan; ^2^ Department of Medical Physics Faculty of Life Sciences Kumamoto University Kumamoto Japan; ^3^ Department of Diagnostic Radiology Graduate School of Medical Sciences Kumamoto University Kumamoto Japan

**Keywords:** radiation reduction, pediatric patient, iterative reconstruction, image noise, temporal bone CT

## Abstract

We performed retrospective (first‐step) and prospective (second‐step) studies to evaluate the body information and noise on temporal bone computed tomography (CT) images in efforts to identify the optimized tube current yielding the greatest reduction in the radiation exposure of pediatric patients undergoing temporal bone CT studies. Our first‐step study included 90 patients subjected to temporal bone CT. We recorded displayed volume CT dose index (${\text{CTDI}}_{\text{vol}}$), displayed dose‐length product (DLP), image noise, and the patient age and sex. We then calculated the optimized tube current value with and without IR corresponding to the children's age based on the ratio of the noise on images from individuals older than 18 years. In our second‐step study, we enrolled 23 pediatric patients and obtained CT scans using our optimized protocol. In both studies we applied identical analysis techniques. The diagnostic image quality was confirmed reading reports and a neuroradiologist. Our first‐step study indicated that the mean image noise in children assigned to five ascending age groups from 2 to 12 years ranged from 167.59 to 211.44 Hounsfield units (HU). In the second‐step study, the mean image noise in each age group was almost the same as the expected noise value and the diagnostic image quality was acceptable. The dose reduction was ranged from 57.5% to 37.5%. Optimization of the tube current–time product allows a radiation reduction without a loss in image quality in pediatric patients undergoing temporal bone CT.

PACS number: 87.57.qp, 87.57.cm

## I. INTRODUCTION

To assess the middle and inner ear congenital hearing deficits, infection, and trauma in children, they are subjected to temporal bone computed tomography (CT).^1^, ^2^, ^3^ Pediatric imaging protocols should be adapted to the size or age of the child to avoid excessive radiation doses.^4^, ^5^ However, at temporal bone CT, size‐dependent tube current adaptation, such as automatic exposure control, is not effective due to the limited scan volume along the Z direction and small variations in the head shape along the angular direction.^(6)^ In addition, unlike at X‐ray radiography, technologist has a difficult task to optimize the age‐appropriate tube current manually because the cross‐sectional area of the head changes by varying age. Furthermore, temporal bone CT requires high resolution, fewer artifacts, and lower noise levels at a thin‐slice thickness of 1.0 mm. Therefore, technologists tend to avoid the substantial reduction of scanning parameters at pediatric temporal bone CT studies regardless of the patient's age. However, it is critical issue how the radiation dose can be reduced without sacrificing image quality in pediatric patients undergoing temporal bone CT.

We retrospectively assessed the body information and noise on temporal bone CT images of patients in different age groups to identify the optimized tube current appropriate for children of different ages. In addition, to lower the tube current we used the iterative reconstruction (IR) technique which permits a greater reduction in the radiation dose than filtered back projection (FBP).^7^, ^8^ We then applied the optimized tube current value in our prospective study of pediatric patients undergoing temporal bone CT, and we discuss the validity of radiation dose reduction and its effect on image quality.

## II. MATERIALS AND METHODS

Our retrospective (first‐step) and prospective (second‐step) studies were approved by our institutional review board; informed patient consent for the analyses was waived for the first‐step, and obtained for the second‐step studies.

### A. First‐step study

#### A.1 Patients and scan technique

In this study, we enrolled 90 patients who underwent temporal bone CT between April 2011 and February 2012. Of these, 46 were children aged 2 to 12 years; the others were adults older than 18 years. To avoid patient number bias in the two age groups, only data obtained before December 2011 were assessed in the adult patients. We carried out a preliminary confirmation of the diagnostic quality of the images that took into account factors recorded in the original readers’ reports, such as image noise and streak and motion artifacts. All temporal bone CT scans were from the superior border of the petrous part to the hemline of the mastoid bone; for image reconstruction we used FBP with an ultra‐high resolution (UHR) kernel. The scanner was a 64‐section CT instrument (Brilliance‐64; Philips Healthcare, Cleveland, OH). The scan parameters were detector configuration, 20×0.625 mm (detector collimation); slice thickness, 0.67 mm; gantry rotation time, 0.75 sec; beam pitch, 0.45; display field‐of view (FOV), 100 mm. The tube voltage and he tube current–time product was 120 kV and 200 mAs, respectively.

#### A.2 Image analysis

We selected 90 images and recorded displayed volume CT dose index (${\text{CTDI}}_{\text{vol}}$), displayed dose‐length product (DLP), image noise, the cross‐sectional head area, and the patients’ age and sex. The cross‐sectional area, measured on scan projection radiographs, was recorded as the product of the anteroposterior and lateral length of the head using the equation:

(1) $$\text{cross}‐\text{sectional area}({\text{cm}}^{2})=(\text{anteroposterior length}\times \text{lateral length})\times \pi /4$$

**Figure 1:**
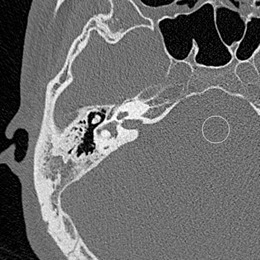
Circular regions of interest (100 mm^2^) were placed in the brain stem region on a slice on which the lateral semicircular canal was clearly observed.

We placed circular regions of interest (ROI) (100 mm^2^) in the brain stem region on the slice in which the lateral semicircular canal was clearly observed (Fig. 1) and measured the image noise expressed as the standard deviation (SD) of the CT value within the ROI. We posited that the radiation dose to pediatric patients was too high because the scanning parameters were the same as adult patients. In our study we divided patients from 2 to 12 years of age (N=46) into four age groups (2–4 years (n=8), 5–7 years (n=18), 8–10 years (n=14), 11–12 years (n=6) (Table 1). We calculated the radiation dose ratio using the ratio of the mean image noise in patients older than 18 years. The detail calculations were as follows: 1) divided pediatric patients into four groups and calculated mean image noise in each group; 2) calculated the decrease in the image noise ratio (dec_noise ratio): dec_noise ratio=mean image noise for each group/mean image noise in patients older than18 years; 3) calculated radiation dose ratio: $\text{reduction dose ratio}=(\text{dec}\_{\text{noise ratio})}^{2}$.

**Table 1:** First‐step study: radiation dose ratio calculated from measurements of the mean image noise in each age group

*Patient Age (yrs)*	*Effective Current–Time Product*	*Mean Image Noise (HU)*	*Decrease in the Image Noise Ratio* ^a^	*Radiation Dose Ratio* ^b^
2−4(n=8)		167.59	0.725	0.526
5−7(n=18)		180.88	0.783	0.613
8−10(n=14)	200	191.36	0.828	0.686
11−12(n=6)		200.92	0.870	0.756
≥19(n=44)		231.05	1.000	1.000

a Decrease in the image noise ratio=Mean image noise for each group/ mean image noise in patients older than18 years.

b $\text{Radiation dose ratio}={\text{Decrease in the image noise ratio}}^{2}$.

### B. Second‐step study

#### B.1 Patients and scan technique

Based on radiation dose ratio, which was decided from first‐step study, from 2 to 12 years we calculated the optimized tube current–time product with and without IR (iDose level 3) (Table 2). In our clinical validation study of the new four‐group protocol, 23 pediatric patients underwent temporal bone CT between July and October 2012. The other scan parameters were as in the first‐step study. We again recorded displayed ${\text{CTDI}}_{\text{vol}}$, displayed DLP, image noise, and the patients’ age and sex.

**Table 2:** Second‐step study: optimal tube current–time product with and without IR reconstruction acquired at 140 kV for each age group

	*Patient Age (yrs)*				
	*2–4*	*5–7*	*8–10*	*11–12*	≥19
Tube current–time product w/o IR (mAs)	105	125	140	155	200
Tube current–time product w. IR^a^ (mAs)	85	100	110	125	160

a The iterative reconstruction enabled a 20% dose reduction.

w/o=without IR; w.=with IR.

#### B.2 Visual inspection of clinical images

For image analysis we used the same techniques as in the first‐step study. The diagnostic quality of the images including image noise and artifacts was confirmed from reading reports. In addition, to improve diagnostic confidence, a radiologist (M.K.) visually re‐inspected the images focusing on the internal acoustic meatus, mastoid air cells, and auditory ossicle including the malleus, incus, and stapes. Visualization on the all images was graded on a four‐point scale where 1=poor (high noise and/or small image with impaired spatial resolution yielding insufficient diagnostic information), 2=fair (image noise partially obscuring the structural contour and/or impaired spatial resolution, degree of diagnostic information acceptable), 3=good (slight image noise and preserved spatial resolution, clarity of the structural contour, degree of diagnostic information sufficient), and 4=excellent (no image noise, preserved spatial resolution, the entire structural contour is smooth and clear, information useful for the diagnosis of middle and inner ear diseases).

#### B.3 Effective dose estimation and dose to the eye lens

We calculated the effective dose for children (aged 5 and 10 years), and for adults (older than 18 years). We estimated the effective dose for each scan by multiplying the displayed DLP value by a standardized conversion factor (${\text{mSv mGy}}^{-1}\;{\text{cm}}^{-1}$):

(2) E=DLP×k(mSv)

where the units of *DLP* is mGy cm and *k* is the region‐specific normalized effective dose conversion factor (${\text{mSv mGy}}^{-1}\;{\text{cm}}^{-1}$). The conversion factor was 0.0057 for patients who were 5 years old, and 0.0042 for 10‐year‐old children; it was 0.0031 for adults undergoing study of the head and neck including the temporal bone.^9^, ^10^ The DLP, provided automatically by the scanner, is the product of ${\text{CTDI}}_{\text{vol}}$ and the length of the exposed volume (i.e., the scan range).

## III. RESULTS

### A. First‐step study

**Figure 2:**
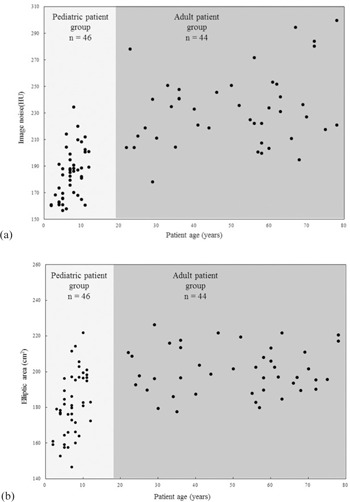
Scatter plots showing the correlation outcomes: (a) relationship between image noise and patient age; (b) relationship between the cross‐sectional area and the patient age.

**Figure 3:**
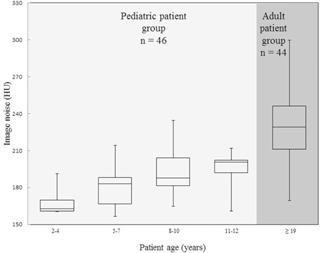
Box‐and‐whisker plot showing the image noise distribution for each age group.

The scatter plot shows the relationship between the patient age and the image noise level (Fig. 2(a)). As shown in Fig. 2(b), like image noise, the cross‐sectional area increased with increasing age. Figure 3 is a box‐and‐whisker plot of the image noise observed for the different age groups. In order of age, at a fixed tube current–time product of 200 mAs, the mean image noise increased from 167.59 to 200.92 HU (Table 1) and had a large value due to the use of UHR reconstruction kernel. Calculation of this value for each age group was based on the assumption that image noise in patients older than 18 years was 231.05 HU. Radiation dose ratio in the 2–4‐, 5–7‐, 8–10‐, and 11–12‐year‐old age groups was 0.526, 0.613, 0.686, and 0.756, respectively (Table 1).

### B. Second‐step study

The optimized tube current–time products with IR were used in the second‐step study. It ranged from 85 mAs (2–4 year olds) to 125 mAs (11–12 year olds) and was 160 mAs in patients older than 18 years; the corresponding dose reduction from 200 mAs was 57.5% and 37.5% for 2–4‐ and 11–12‐year‐old patients. Mean image noise in the 2–4, 5–7, 8–10, and 11–12 years age groups was 193.06, 194.85, 205.60, and 172.18 HU, respectively (Table 3), and were close to expected image noise level for adult patients of 201.01 HU. Mean visualization score of the internal acoustic meatus, mastoid air cells, and auditory ossicle assigned by the radiologist was 3.0, 2.96, and 2.93, respectively. In most patients visualization was scored as good.

**Table 3:** Second‐step study: image noise at different tube current–time product in each age group

*Patient Age (yrs)*	*Tube Current–Time Product with iDose (mAs)*	*Mena DLP (mGy cm)*	*Mean Image Noise (HU)*
2−4(n=9)	85	122.37	193.06
5−7(n=6)	100	148.67	194.85
8−10(n=5)	110	175.94	205.6
11−12(n=3)	125	201.7	172.18

### C. Effective dose

The mean effective dose for patients at 5 years old and 10 years old was 0.85 and 0.75 mSv (Table 4). The values were almost the same as adult even though the dose reduction was decreased by 50.0% and 45.0% for 5‐ and 10‐year‐old patients. This is due to the fact that k factors for patients at 5 years old and 10 years old were 1.84 and 1.35 times larger than that for adult patients.

**Table 4:** First‐ and second‐step studies: mean effective dose in the different age groups

		*5 Years Old*	*10 Years Old*	*Adult*
$k({\text{mSv mGy}}^{-1}\;{\text{cm}}^{-1})$		0.0057	0.0042	0.0031
Mean effective dose (mSv)	Pre‐	1.82	1.38	1.12
	Post‐	0.85	0.75	0.84

## IV. DISCUSSION

In this study we focused how radiation dose could be reduced in pediatric temporal bone CT. We reviewed ${\text{CTDI}}_{\text{vol}}$, DLP, image noise, and the age and sex of 90 patients included in the first‐step investigation. We then established an optimized pediatric scanning protocol and performed a second‐step study in which temporal bone CT images were evaluated for their diagnostic quality, despite a reduction in the radiation dose delivered to 23 patients separated into specific age groups.

In the first‐step study, the image noise was increased with increasing the age of pediatric patients (2–12 years). Image noise is affected by the head size. Huda et al.^(11)^ reported that the head size increases markedly during the first two years of life and then increases gradually until the age of around 18 years. Based on our findings with respect to the image noise, we divided patients younger than 12 years into four groups. We then designed a protocol in which the image noise level was the same as in adults (231.05 HU, Table 2), expecting that the noise level in the second‐step study would be similar regardless of the patients’ age. We achieved a further reduction in image noise by applying iDose at level 3. Using the iDose level 4 at temporal bone CT in adults, Niu et al.^(10)^ achieved a radiation dose reduction of 50% compared to routine protocols with FBP; diagnostic image quality was maintained. As at level 3, iDose decreases the image noise to 0.78 of the original level; at a 20% radiation reduction, it increased the image noise to 1.12 of the original level. Therefore, total decreasing noise rate is 0.87 (0.78×1.12) and the expected image noise level corresponds to 201.01 HU (231.05×0.87). In our second‐step study, mean image noise for pediatric patients was 191.43 HU, revealing a good relationship between our expected image noise level at 201.01 HU and second‐step data. In addition, the image quality was diagnostically acceptable even though the tube current–time product was changed.

Radiation dose reduction is a key concern in pediatric CT studies.^9^, ^12^, ^13^, ^14^ In addition, in the optimization of scan protocols and the acquisition of reliable risk assessments, the radiation doses delivered to individual organs must be considered. Pearce et al.^(15)^ reported that in children, radiation dose exposure from head CT raises certain health risks. Assuming the delivery of typical doses at scans performed after 2001 in children younger than 15 years, the cumulative ionizing radiation dose from two to three head CT studies (i.e., ∼60 mGy) may triple the risk for brain tumors and five to ten head CT studies (∼50 mGy) triple the risk for leukemia. Because children are more highly radiosensitive than adults, the stochastic effects of the radiation dose and the effective dose also tend to be higher than in adults.^16^, ^17^, ^18^

The Image Gently website (http://www.imagegently.org) lists universal protocols and is a resource for medical professionals involved in the care of children. It facilitates the establishment of head CT protocols and the calculation of the appropriate tube current–time product for pediatric patients of varying sizes. The ratio of the tube current–time product vis‐à‐vis adults is 0.74 for infants, 0.86 for 2‐, and 0.93 for 6‐year‐old patients. In our study, it was 0.53 for 2–4‐ and 0.61 for 5–7‐year‐old patients undergoing temporal bone CT (see Table 1). Unlike that of the abdomen, the head size varies little between Asian and Western individuals. Consequently, the findings reported here may be of value not only to our institution, but also to others performing pediatric temporal bone CT studies, and may facilitate a reduction in the radiation exposure of these patients.

Our study has some limitations. First, because we included only patients with diagnostically acceptable images acquired at a fixed tube current–time product of 200 mAs, at 90 the number of patients included in our first‐step study may be relatively small. In addition, pediatric patients for second‐step study were also small numbers and we need further validation. Second, although we represented the ratio of tube current–time product with increasing age, the information should be carefully applied to other CT models of the same manufacture or to CT equipment of other manufactures. Third, because one radiologist inspected the diagnostic confidence of the images in first‐ and second‐step study, we could not evaluate intra/interobserver agreement. Finally, because we did not obtain the scanning data with fixed 200 mAs for under 2 years old, we excluded the tube current–time product for those under 2 years old.

## V. CONCLUSIONS

We optimized the tube current–time product for different pediatric ages based on the image noise on temporal bone CT images of adults. We found that the radiation dose can be reduced while maintaining the quality of images obtained at pediatric temporal bone CT.
